# Ectopic intramural pregnancy developing at the site of a cesarean section scar: a case report

**DOI:** 10.1186/1757-1626-2-9404

**Published:** 2009-12-30

**Authors:** Adel Al-Nazer, Lama Omar, Manal Wahba, Tahrir Abbas, Mariam Abdulkarim

**Affiliations:** 1 Department of Obstetrics and Gynocology Dubai Hospital, Dubai, UAE

## Abstract

**Introduction:**

Cesarean section scar pregnancy is a rare but serious complication. It occurs in women with previous uterine scar when implantation takes place at the site of this scar. If early diagnosis is missed the woman's future fertility and eve her life are at risk. Earlier reports on the condition suggesting different management approaches have been described.

**Case presentation:**

A 37 year-old woman gravida 4, para 3, was referred to our emergency department, as a case of missed miscarriage following 14 weeks amenorrhea. Ultrasound examination revealed a picture suggestive of intramural pregnancy near the Cesarean section scar. The case was managed by laparotomy evacuation of products of conception and repair of the scar.

**Conclusion:**

The diagnosis of ectopic intramural pregnancy in a cesarean section scar is possible with ultrasound and high level of suspicion. This serious complication must be suspected in a pregnant woman with previous uterine scar when early ultrasound show a gestational sac that is implanted anteriorly in the lower uterine segment, near the uterine scar. Ultrasound criteria for diagnosis include empty uterus, empty cervical canal and a discontinuity on the anterior wall of the uterus demonstrated on a sagittal plane of the uterus running through the amniotic sac. Early intervention is recommended to avoid serious consequences in such cases.

## Introduction

Pregnancy in a Cesarean Section scar (CSS pregnancy) is a rare complication that may have serious consequences affecting the woman's future fertility and may even affect her own life. It is believed to develop as a result of the presence of a microscopic tract in the scar allowing the blastocyst to be implanted deep in the myometrium [1].

Early diagnosis of CSS pregnancy is essential to avoid serious complication such as sever hemorrhage which may require hysterectomy and endanger the woman's' life. Diagnosis is based on high level of suspicion when a gestational sac is seen at sonography on the anterior lower part of the uterus in a woman with a previous Cesarean section [1].

The differential diagnosis includes spontaneous abortion and cervico-isthmical pregnancy. It is essential to get a proper history and a high resolution ultrasound scanning in order to avoid wrong diagnosis [2].

Treatment modalities are diverse with no one option being superior to others. Most reported cases are sporadic and subject of trials.

Surgical treatment by evacuation of the sac, excision and repair of the gaped scar through laparotomy [3] or laparoscopy [4] has been tried. Dilatation and evacuation was also reported [5]. Medical treatments using local [1] or systemic Methotrexate (MTX) [6] have been used frequently. Potassium chloride with or without MTX was also reported [7]. Uterine artery embolisation was also used in combination with either medical or surgical approaches [8]. A combination of surgeries and medical treatment was also tried. There is no optimum line of treatment that can be universally recommended and each case should be treated individually.

## Case presentation

**Figure 1:**
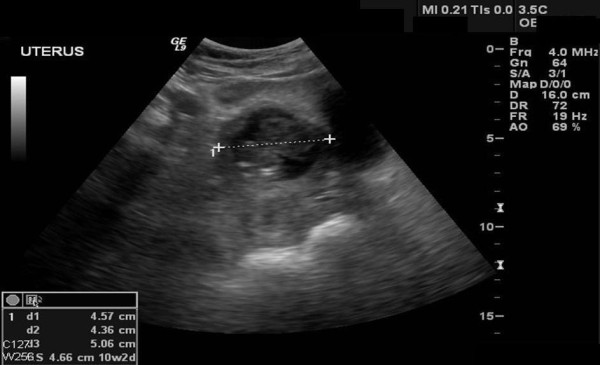
**Abdominal ultrasound, transverse scan showing a gestational sac measuring 4.57 × 4.36 cm between the uterus and the urinary bladder**.

**Figure 2:**
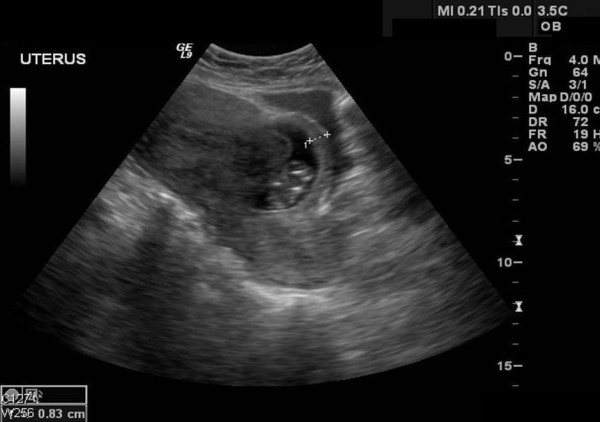
**Abdominal ultrasound, longitudinal scan showing a gestational sac pushing itself towards the urinary bladder**. An 8 mm layer of fibro-muscular tissue separates the sac from the bladder.

A 37 year-old United Arab Emaerite woman gravida 4, para 3, was referred to our emergency department, as a case of missed miscarriage following 14 weeks amenorrhea. The patient's obstetric history included three Cesarean sections last one 9 years back. The first Cesarean section was done at 7 month gestation due to placental abruption, the second was done for placenta previa and the third was done electively. The patient presented with history of lower abdominal pain and bleeding per vagina for the last 12 days. Vaginal examination revealed an enlarged uterus of 8 weeks size that is slightly tender on movement. The cervical os was closed with no vaginal bleeding. Trans-abdominal ultrasound in transverse scan (Figure 1) shows enlarged uterus, empty uterine cavity and cervical canal and a gestational sac (4.57 × 4.36 cm) situated in the lower part of the anterior uterine wall at the site of the Cesarean section scar. In a longitudinal scan (Figure 2) the sac is seen protruding through the anterior uterine wall and pushing towards the bladder with a thin layer of myometrium (0.8 cm thick) separating them. A non viable embryonic echo was observed inside the sac measuring 3.5 cm at Crown Rump Length (CRL) corresponding to 10 weeks plus three day gestation. No fluid was seen in the cul-de-sac. With these ultrasound criteria present an ectopic implantation in the previous Cesarean section scar was considered. The mother was counselled regarding the treatment options, but due the possibility of prolonged follow up period with medical treatment she preferred to go for surgical intervention.

**Figure 3:**
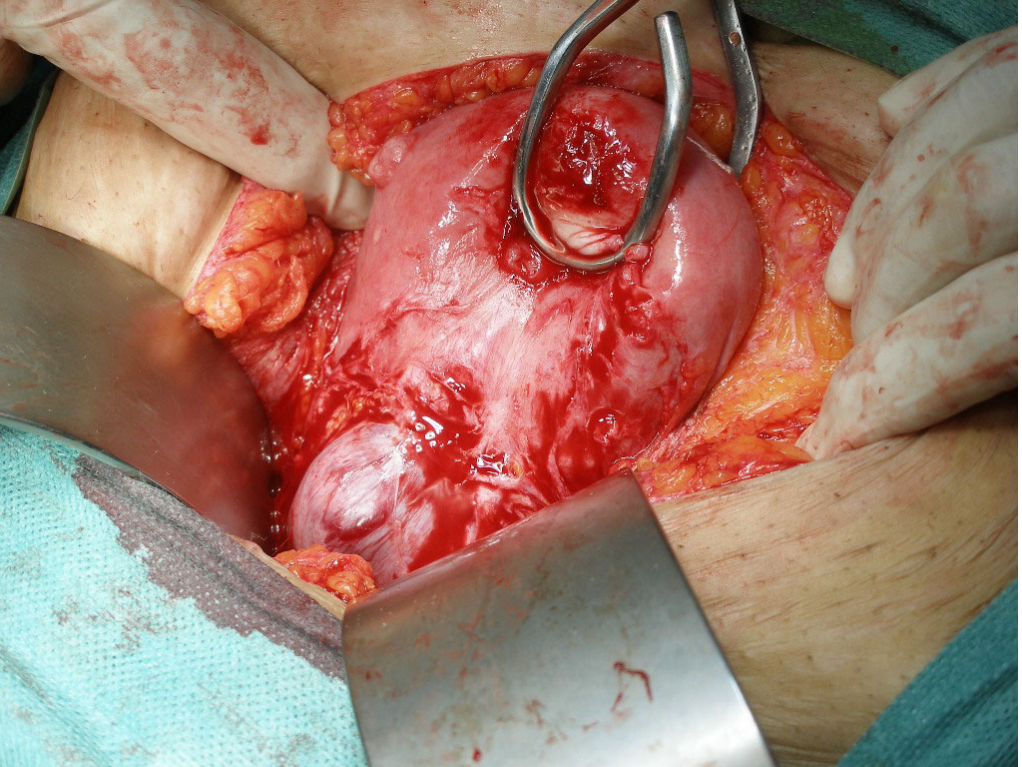
**Operative view a gestational sac bulging into the anterior uterine wall after dissection of the urinary bladder**.

**Figure 4:**
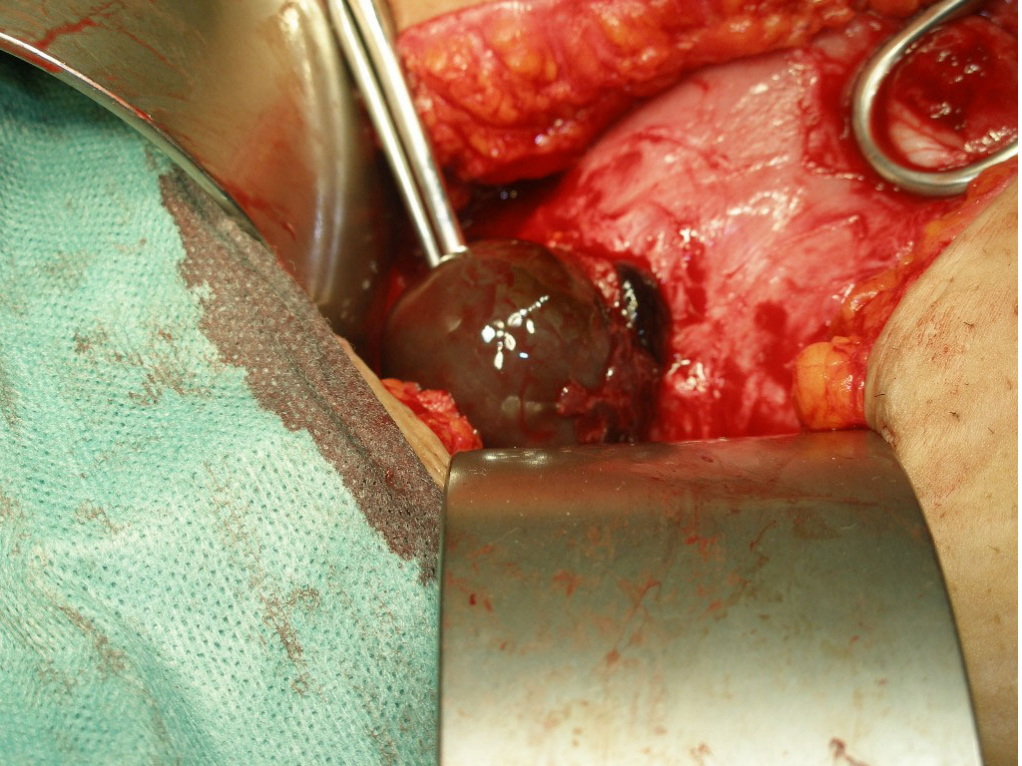
**Operative view the bluish gestational sac bulging out of the dehiscent scar after the fibro-muscular layer was gently incised**.

A laparotomy was performed with Pfannenstiel incision under general anesthesia. The bladder was adherent to the lower part of the uterus so the peritoneum was incised and the bladder was dissected down to the cervix. The gestational sac was seen bulging and thinning out the uterine wall anteriorly at the scar site (Figure 3). Most of the scar was in fact disrupted except for a thin layer of fibro-muscular tissue. When this layer was gently incised the sac bulged out with its bluish color (Figure 4). Approximately 4-5 cm intact gestational sac was delivered followed by a placental tissue. The implantation of the ectopic pregnancy caused dehiscence of the previous Cesarean section scar. A trial to identify the cervical canal by retrograde probing was not easy as there was a thin layer of uterine tissue separating the implantation site from the uterine cavity. When this layer was overcome a communication was created between the implantation site and the uterine cavity. The dehiscent scar was then repaired, haemostasis was assured and bilateral tubal ligation was done according the request of the patient and her husband. The estimated blood loss was less than 500 ml and there was no need for blood transfusion. The patient had an uneventful postoperative recovery and was discharged from the hospital on postoperative day 5.

## Discussion

The increasing number of Cesarean Sections in recent years has brought into light a set of complications that were not so frequently observed in the past. Intramural implantation through a Cesarean scar is a rare complication that is diagnosed with increasing frequency in recent years. Larsen & Solomom reported the first case in 1978 as a post-abortal haemorrhage due to what they described as uterine scar saculus [9]. Since then cases have been reported with increasing frequency leading to better understanding of the condition. The possible incidence of this abnormality ranges from 1:1800 to 1:2200 pregnancies [2].

It is possible that this complication is related to the poor healing of the cesarean section scar and the implantation of the gestational sac into it. It may also result from a defect in the endometrium caused by trauma created by minor procedures as embryo transfer in the assisted reproduction techniques [10]. The natural history of this abnormal implantation is not clear but it may result in a pregnancy that grow towards the uterine cavity and looses its vascular connection causing spontaneous abortion. Or it may continue to grow to near term gaining new stronger vascular connections ending into a low-lying adherent placenta with or without invasion of surrounding organs. If the gestational sac grows away from the uterine cavity and pushes towards the bladder this may result what is referred to in the literature as Cesarean Scar Pregnancy. This abnormal pregnancy can't continue to term, and if left to grow it end in rupture and massive hemorrhage and may endanger the woman's life. Early diagnosis by ultrasonography is thus important to avoid serious consequences. The sonographic criteria essential for diagnosis are (i) Empty uterus (ii) Empty cervical canal (iii) Development of the sac in the anterior part of the isthmic portion and (iv) A discontinuity on the anterior wall of the uterus demonstrated on a sagittal plane of the uterus running through the amniotic sac. Another important ultrasound criterion is absent or diminished healthy myometrium between the bladder and the sac [11]. Also Jurkovic et al proposed Doppler examination that shows high velocity with low impedance peri-trophoblastic vascular flow clearly surrounding the sac [12].

Although most reported cases are misdiagnosed in the beginning for miscarriage yet with high level of suspicion and with the aid of ultrasonography the two conditions can be easily differentiated. Cervicoisthmical pregnancy is another rare condition that can be a source of confusion. This pathological condition may be very similar to CSS pregnancy. The differentiating points include the absence of healthy uterine tissue between the sac and the bladder [11]. Another differentiating sign is the absence of "sliding organ sign" which is defined as the inability to displace gestational sac from its position at the level of the internal os using gentle pressure applied by the transvaginal probe [13].

In this case report we present a CSS pregnancy that was misdiagnosed as missed abortion. This confusion is common and most cases need repeated scanning before the diagnosis is reached. When the patient was referred to us at 10 weeks size (14 weeks amenorrhea) the ultrasound picture was already clear.

The challenge in diagnosis is further complicated by a challenge in treatment. Due to the relative rarity of the condition there are no optimal lines for therapy. Treatment modalities are either medical or surgical. The medical treatment consists of administration of MTX locally [12] or systemically [6]. Others combine Potassium Chloride injected locally into the fetal thorax with MTX injected into the sac [13]. The medical treatment requires prolonged follow up for complete resolution of the ectopic pregnancy. In many instances bleeding may commence following the medical treatment which may require surgical intervention. Failure of pregnancy resorption and persistence of a relatively large gestational sac may also require intervention. Medical treatment also has many side effects more with the systemic than with the local rout. Another important issue is the condition of the uterine scar left after medical treatment and its subsequent behavior in future pregnancies. Hasegawa et al reported dehiscence in some cases after successful medical treatment with local MTX and a repeated scar pregnancy. They believed that excision of the old scar and repair of its site could reduce the risk of dehiscence and recurrence [14].

The surgical approach involves the traditional laparotomy and evacuation of the sac and repair of the uterine defect [11]. The same procedure can be done laparoscopically [4]. Dilatation of the cervix and evacuation has also been described by some with apparently good outcome [5]. This does not look to be a rational approach as the gestational sac is usually separated from the uterine cavity by a layer of myometrium and an attempt to evacuate the sac vaginally will result in unavoidable tearing of this layer with possible excessive bleeding. Graesslin et al reported successful use of systemic MTX prior to dilatation and curettage in one case [15]. Some authors prefer to use uterine artery embolization (UAE) in order to minimize blood loss. Yan reported four cases in three of them UAE was used either following systemic or before local administration of MTX [8]. Although UAE appears promising in treating stable cases, it is too early to be recommended as a primary line of therapy.

It is important however to emphasize that due to the lack of enough available data no treatment modality can guarantee uterine integrity or can be promoted as optimal [12]. Treatment thus must be individualized according to the sac size, presence of fetal heart, BHCG level, the desire for future fertility and the experience and facilities available.

In our case although her condition was stable yet laparotomy was selected as a line of management as the mother did not want to have a prolonged follow up period. Although the pregnancy was nonviable and expectant management by follow up with BHCG was possible, the size of the gestational sac and the woman's refusal of a long follow up period justified the active approach.

Counseling patients with CSS pregnancy is difficult. There is no enough data regarding the optimum treatment or a guideline for management. With the accumulation of reported cases it may be soon possible to have a guideline for optimum management as it is now clear regarding diagnosis.

## Conclusion

The incidence of Cesarean Section is increasing year after year. The complications of this mode of delivery are not limited to the time of surgery. Remote complications especially in subsequent pregnancies are also of significance. Cesarean Section scar pregnancy is also is one of these possible complications that may lead to rupture of the uterus, bleeding and may endanger the woman's life. Early diagnosis is possible with high level of suspicion and with the aid of high quality ultrasound scanning. The appropriate treatment of such cases is not yet clear. The accumulation of data may help to understand the disease better and to develop the appropriate plan of management.

## Consent

Written informed consent was obtained from the patient for publication of this case report and accompanying images. A copy of the written consent is available for review by the Editor-in-Chief of this journal.

## Competing interests

The authors declare that they have no competing interests.

## Authors' contributions

AA analyzed and interpreted the patient data and contributed in preparation of the manuscript. LO performed the ultrasound. MW assisted in the ultrasound and in the preparation of the manuscript. TA was involved in the imaging preparation and reviewed the manuscript. MA reviewed the literature and participated in reviewing the manuscript. All authors read and approved the final manuscript.
